# Supplementary material for: Dithioethanol (DTE)-Conjugated Deoxyribose Cyclic Dinucleotide Prodrugs (DTE-dCDNs) as STING Agonist
Source: Int J Mol Sci. 2023 Dec 20;25(1):86. doi: 10.3390/ijms25010086 (PMC10778758; doi:10.3390/ijms25010086)

Supporting Information

Dithioethanol (DTE)-Conjugated Deoxyribose Cyclic Dinucleotide Prodrugs (DTE-dCDNs) as STING Agonist

Zhiqiang Xie [1], Yuchen Yang [1], Zhenghua Wang [1], Dejun Ma [1] and Zhen Xi [1,2,*]

[1] State Key Laboratory of Elemento-Organic Chemistry, Department of Chemical Biology, College of Chemistry, Nankai University, Tianjin 300071, China; 1120200432@mail.nankai.edu.cn (Z.X.); 2120200933@mail.nankai.edu.cn (Y.Y.); wzhnren@163.com (Z.W.); madejun@nankai.edu.cn (D.M.)
[2] Frontiers Science Center for New Organic Matter, Nankai University, Tianjin 300071, China
* Correspondence: zhenxi@nankai.edu.cn

Table of Contents

Title	Page
Table S1. RT-qPCR primers of target genes and the internal control gene	S2
Figure S1. UPLC-MS spectra for the mixture of the prodrug **9** and GSH	S3
Table S2. The half-life period of prodrug **9** and CDNs	S3
Figure S2. Prodrug **9** did not stimulate the IFN-β gene transcription in CT-26 cells	S4
Figure S3. Prodrug **9** stimulated the production of IFN-β in THP-1 cells	S4
Figure S4. Relative quantification of phospho-STING, phospho-TBK1, phospho-IRF3	S5
Chemical characterization of ^{1}H, ^{13}C, ^{31}P NMR and HPLC spectra	S6-S15
Raw Images of western blots	S16-S22

Table S1. RT-qPCR primers of target genes and the internal control gene

Primer name	Sequence (5’-3’)
IFN-β-F	AACAAGTGTCTCCTCCAAAT
IFN-β-R	TCTCCTCAGGGATGTCAAAG
CXCL10-F	CATTCTGATTTGCTGCCTTAT
CXCL10-R	TTGATGGCCTTCGATTCTGG
IL-6-F	AGACAGCCACTCACCTCTTCAG
IL-6-R	TTCTGCCAGTGCCTCTTTGCTG
TNF-α-F	TGAAAGCATGATCCGGGACG
TNF-α-R	AGGCAGAAGAGCGTGGTGGC
GADPH -F	GGACCTGACCTGCCGTCTA
GADPH-R	GAGTGGGTGTCGCTGTTGA

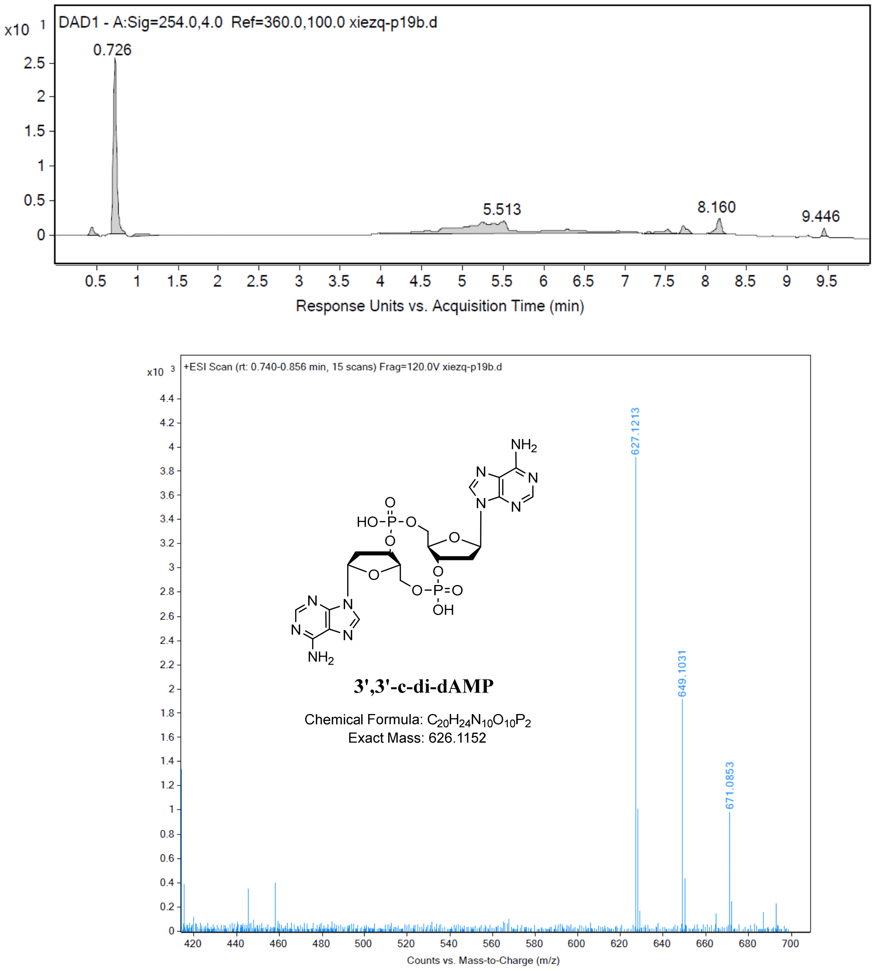

Figure S1: UPLC-MS spectra for the mixture of the prodrug **9** and GSH for 0.5 h at 37°C.

Table S2. The half-life period of prodrug **9** and CDNs

Compounds	$T_{1/2}$（h）
**9**	28
3′,3′-c-di-dAMP	>72
2',3'-cGAMP	31
ADU-S100	67

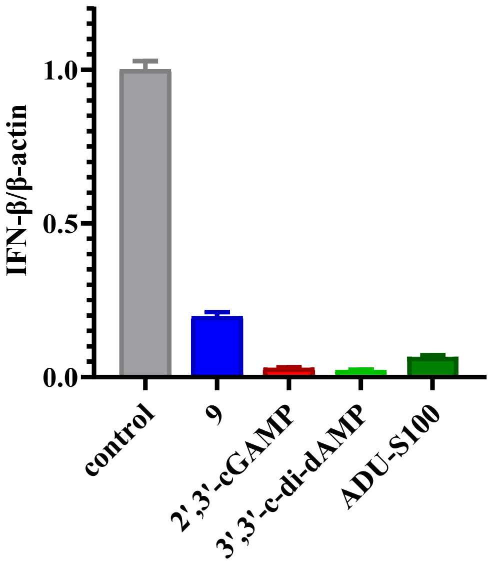

Figure S2. Prodrug **9** did not stimulate the *IFN-β* gene transcription in CT-26 cells. The mRNA expression levels of *IFN-β* were assessed by real-time quantitative PCR (qPCR) under the treatment of prodrug **9** (10 μM), 3′,3′-c-di-dAMP (100 μM), 2′,3′-cGAMP (100 μM) and ADU-S100 (10 μM) for 4 h.

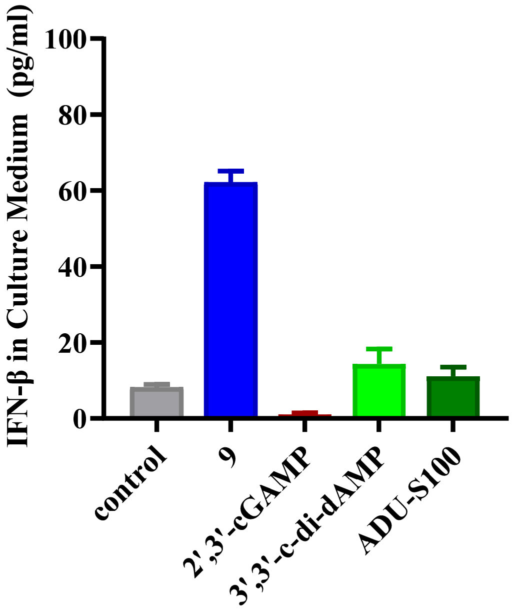

Figure S3. Prodrug **9** stimulated the production of IFN-β in THP-1 cells. Under the treatment of prodrug **9** (10 μM), 3′,3′-c-di-dAMP (100 μM), 2′,3′-cGAMP (100 μM) and ADU-S100 (10 μM) for 4 h, the levels of IFN-β were assessed by an enzyme-linked immunosorbent assay (ELISA).

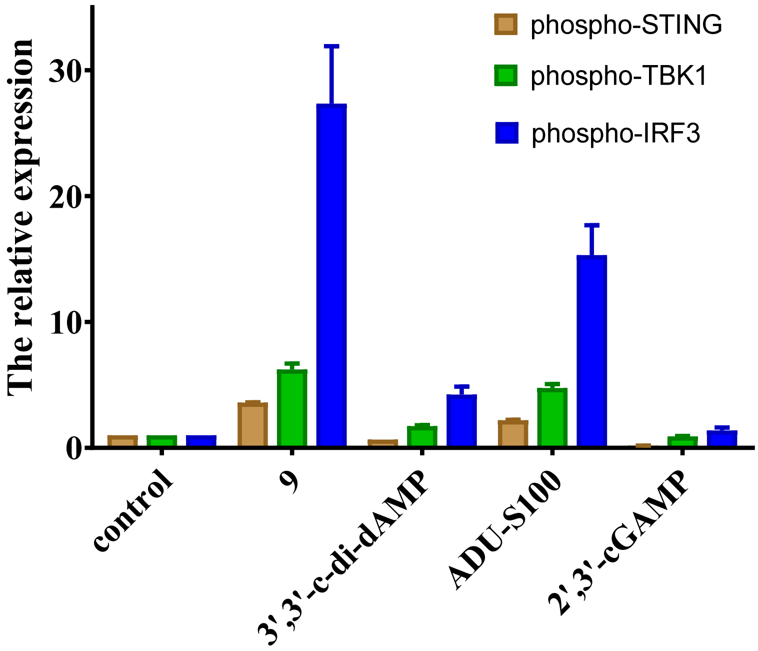

Figure S4. Relative quantification of phospho-STING, phospho-TBK1, phospho-IRF3. Prodrug **9** (10 μM), 2′,3′-cGAMP (100 μM), 3′,3′-c-di-dAMP (100 μM) and ADU-S100 (10 μM) were used to treat THP-1 cells for 4 h, and the levels of total STING, phospho-STING, total TBK1, phospho-TBK1, total IRF3, phospho-IRF3, and β-actin were assessed by western blotting.

Chemical characterization of ^{1}H, ^{13}C, ^{31}P NMR and HPLC spectra

^{1}H, ^{13}C, ^{31}P NMR spectra of **3**:

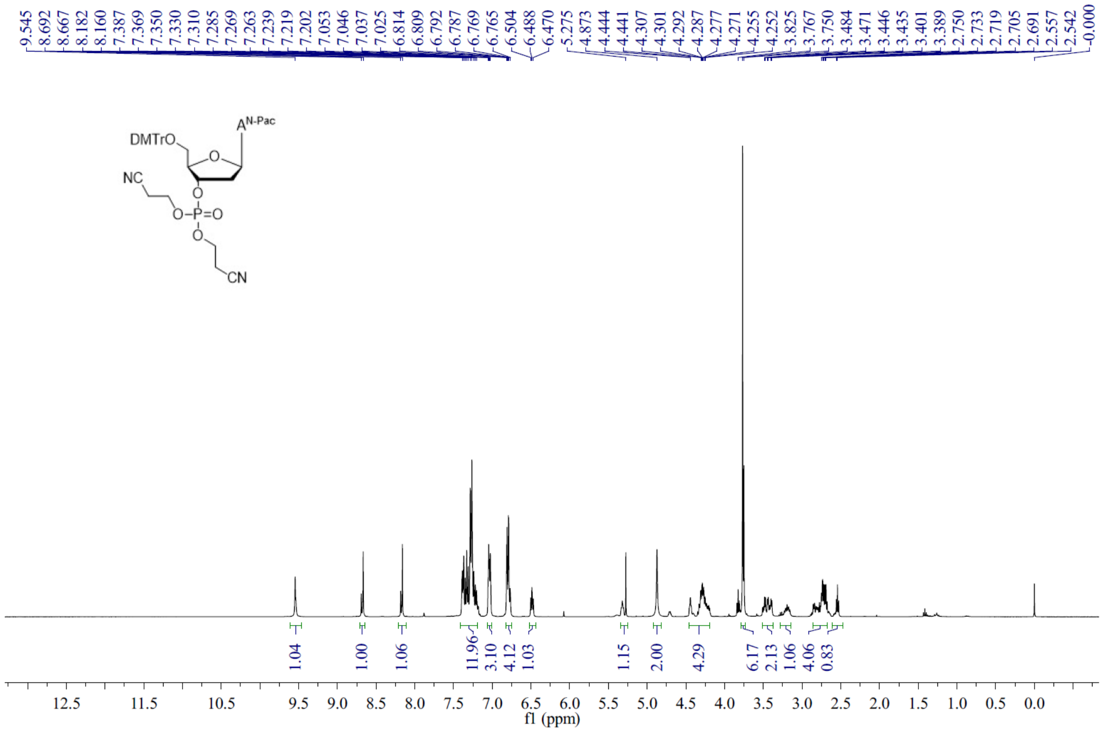

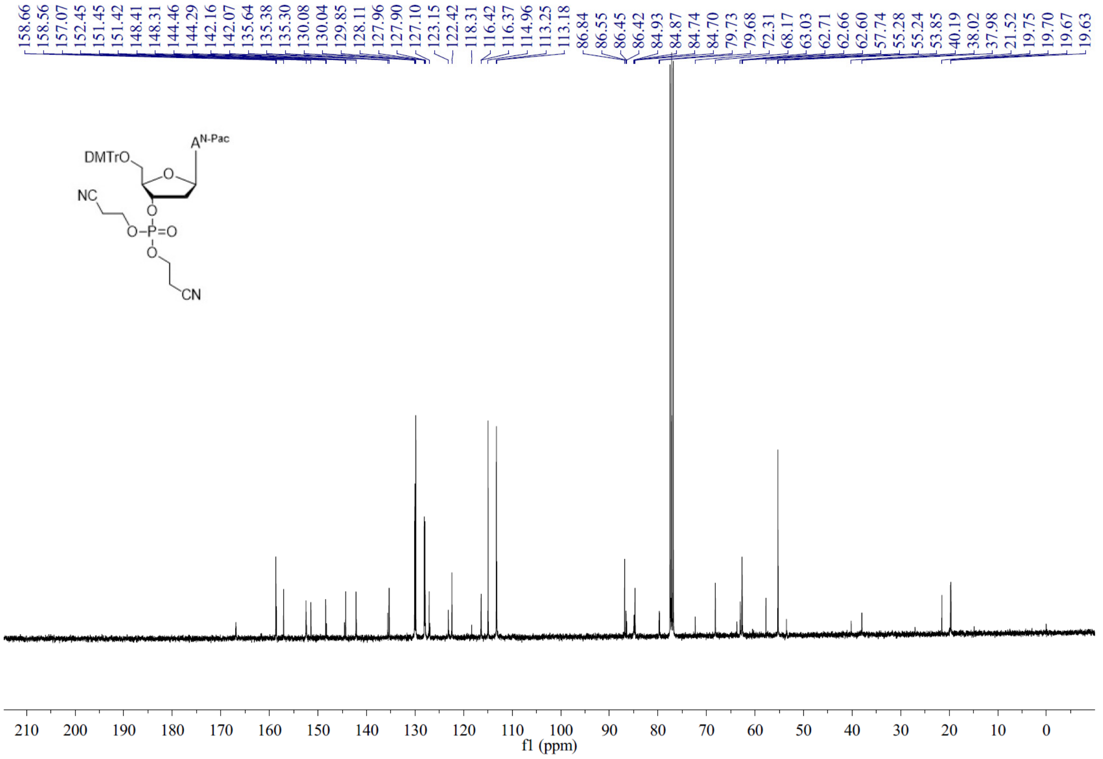

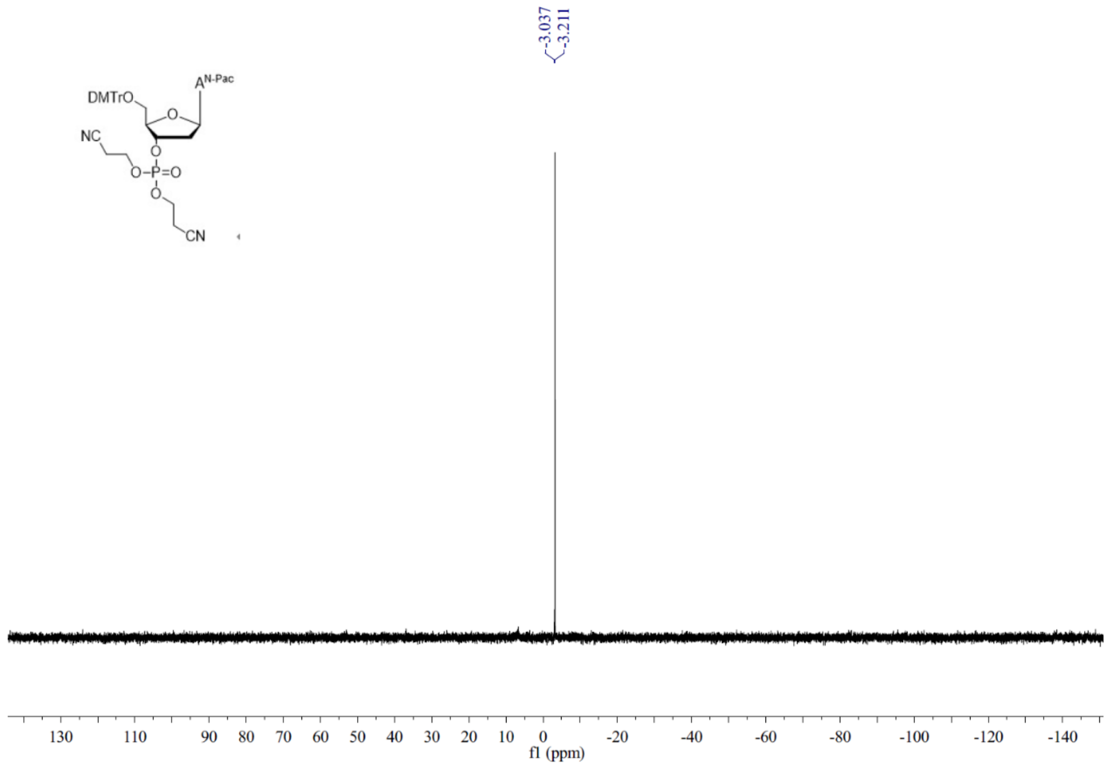

^{1}H, ^{13}C, ^{31}P NMR spectra of **4**：

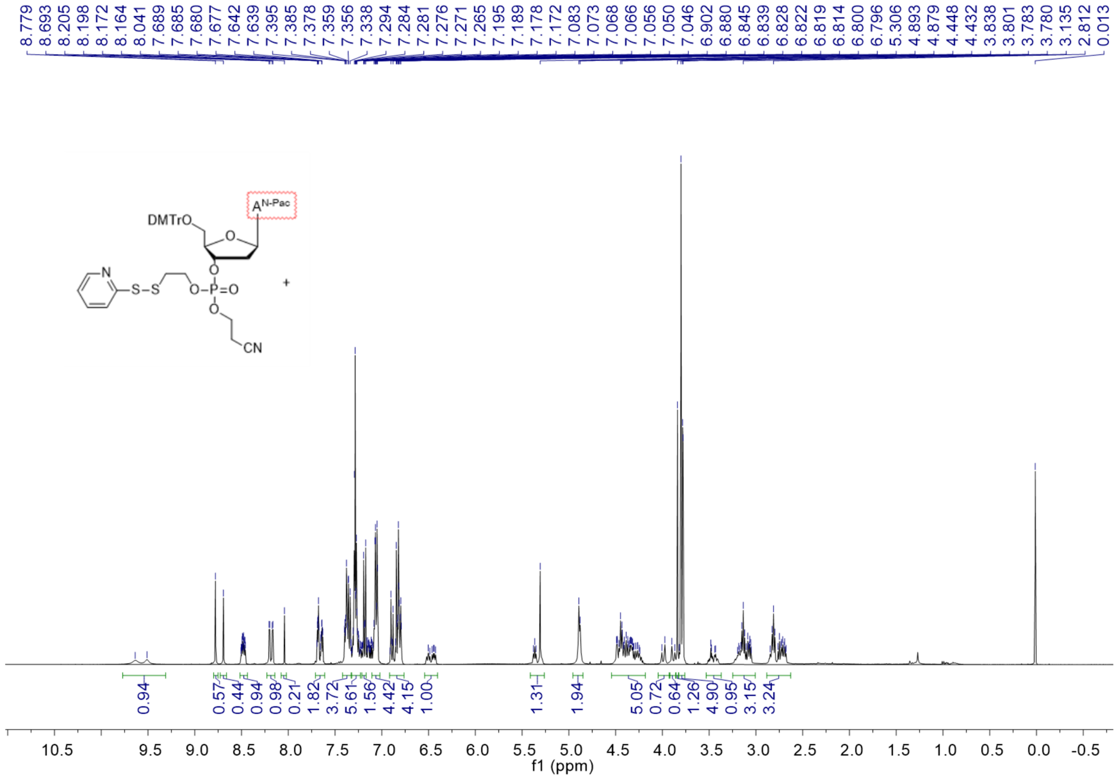

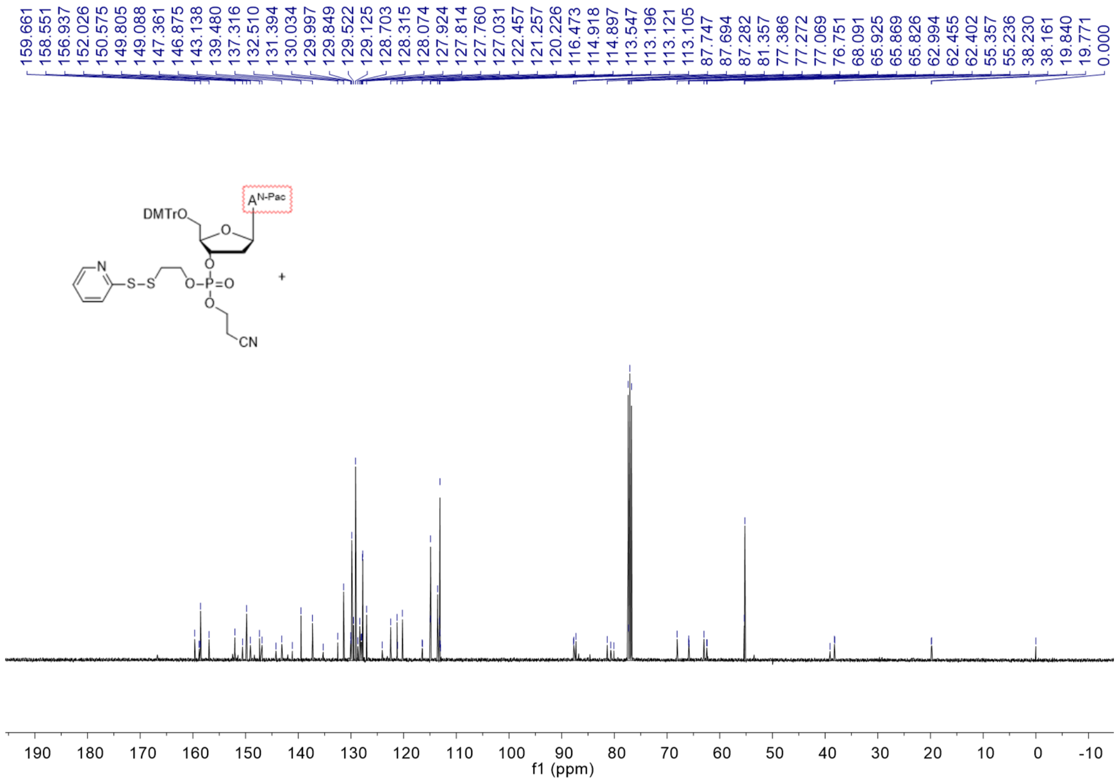
159.661
158.551
156.937
152.026
150.575
149.805
149.088
147.361
146.875
143.138
139.480
137.316
132.510
131.394
130.034
129.997
129.849
129.522
129.125
128.703
128.315
128.074
127.924
127.814
127.760
127.031
122.457
121.257
120.226
116.473
114.918
114.897
113.547
113.196
113.121
113.105
87.747
87.694
87.282
81.357
77.386
77.272
77.069
76.751
68.091
65.925
65.869
65.826
62.994
62.455
62.402
55.357
55.236
38.230
38.161
19.840
19.771
0.000
DMTrO
A^N-Pac
O
N
S–S
O–P=O
CN
+
190 180 170 160 150 140 130 120 110 100 90 80 70 60 50 40 30 20 10 0 -10
f1 (ppm)

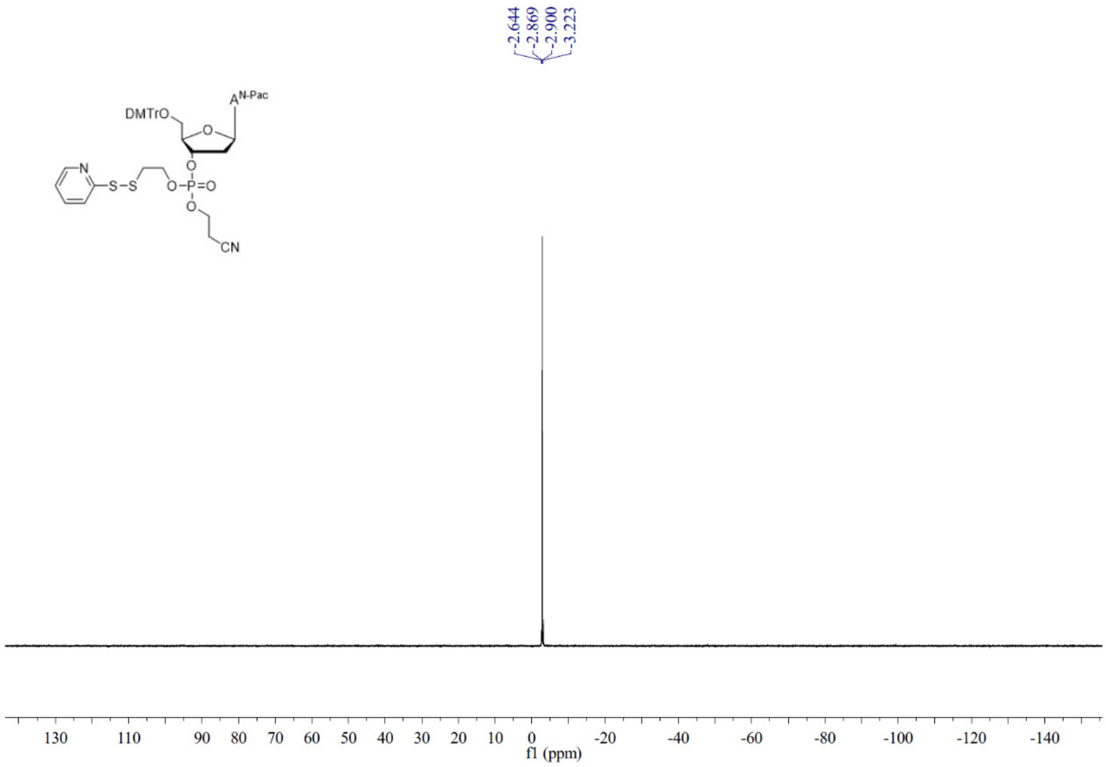
-2.644
-2.869
-2.900
-3.223
DMTrO
A^N-Pac
O
N
S–S
O–P=O
CN
130 110 90 80 70 60 50 40 30 20 10 0 -20 -40 -60 -80 -100 -120 -140
f1 (ppm)

^{1}H, ^{13}C, ^{31}P NMR spectra of **6**:

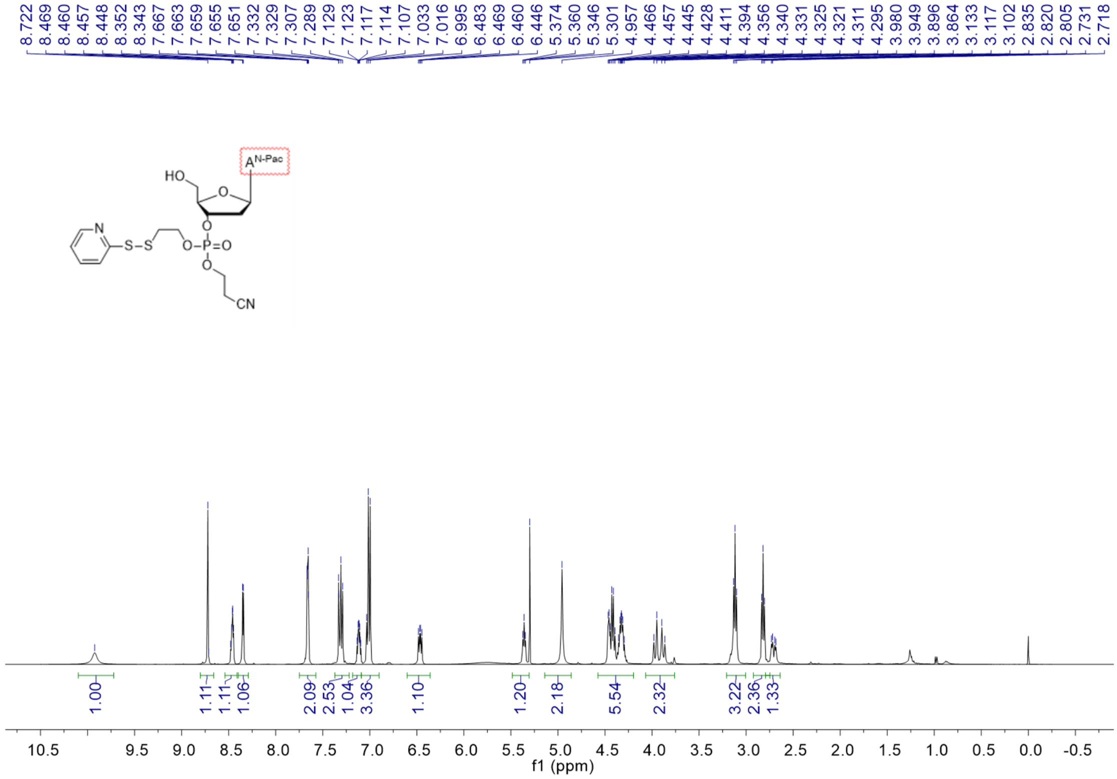

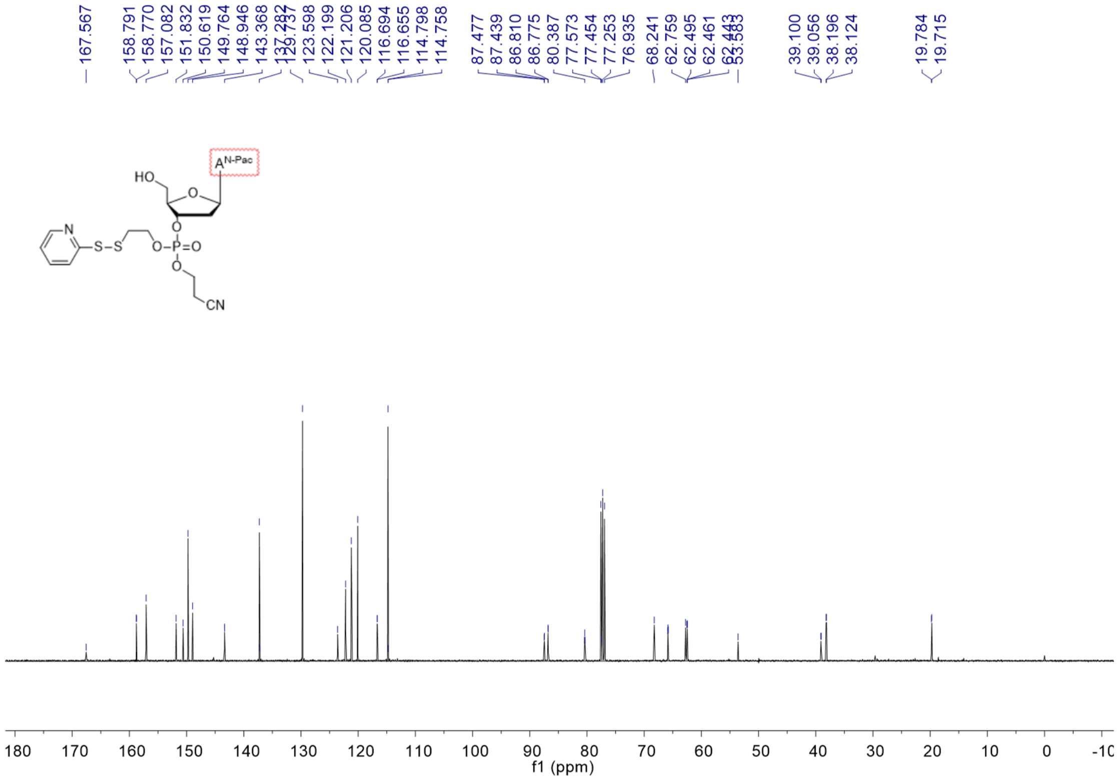

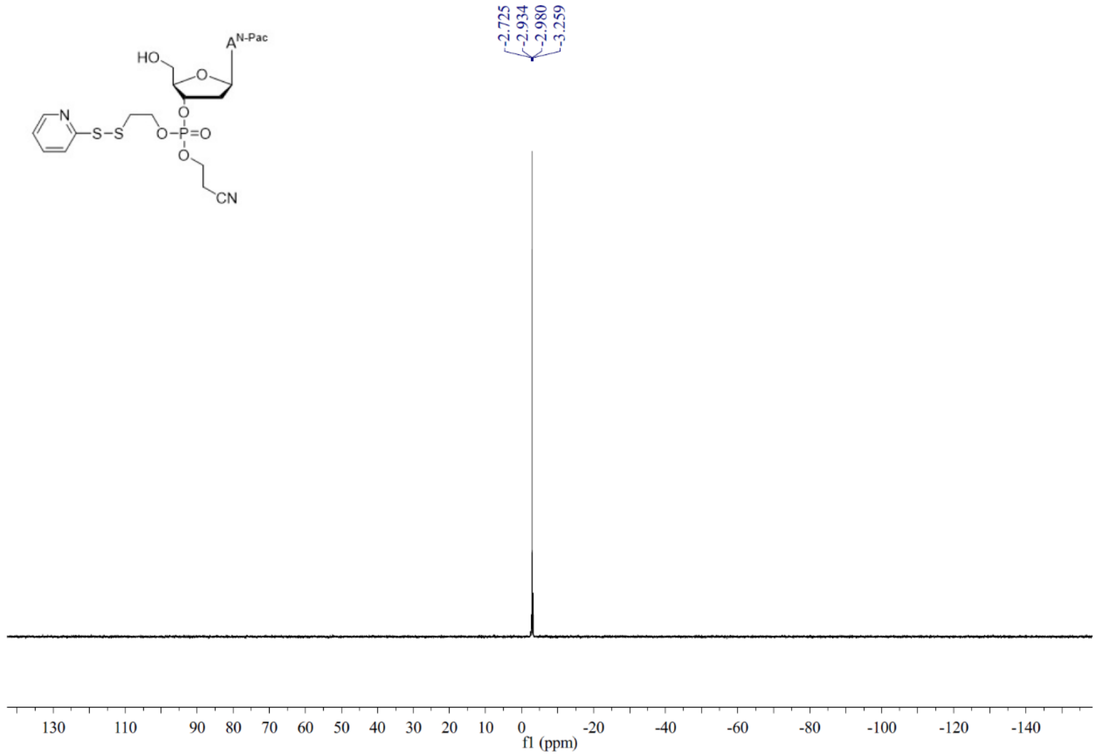

^{1}H, ^{13}C, ^{31}P NMR spectra of **7**：

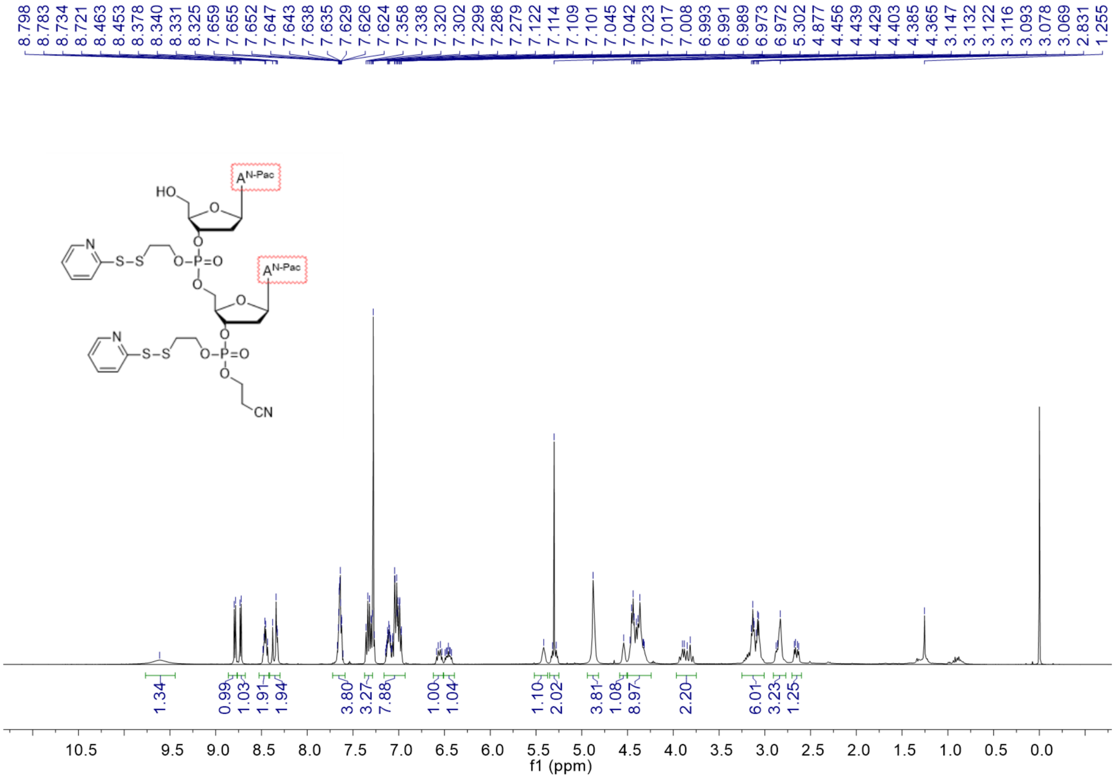

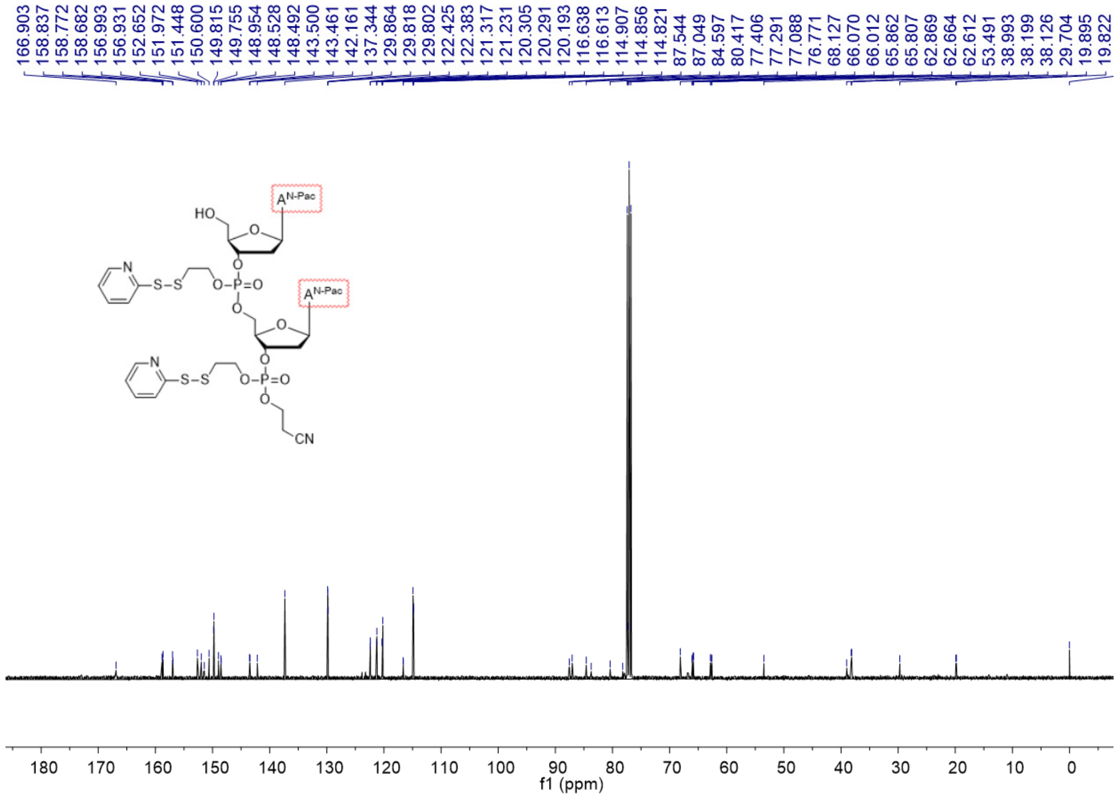
166.903
158.837
158.772
158.682
156.993
156.931
152.652
151.972
151.448
150.600
149.815
149.755
148.954
148.528
148.492
143.500
143.461
142.161
137.344
129.864
129.818
129.802
122.425
122.383
121.317
121.231
120.305
120.291
120.193
116.638
116.613
114.907
114.856
114.821
87.544
87.049
84.597
80.417
77.406
77.291
77.088
76.771
68.127
66.070
66.012
65.862
65.807
62.869
62.664
62.612
53.491
38.993
38.199
38.126
29.704
19.895
19.822
HO
A^N-Pac
CN
f1 (ppm)

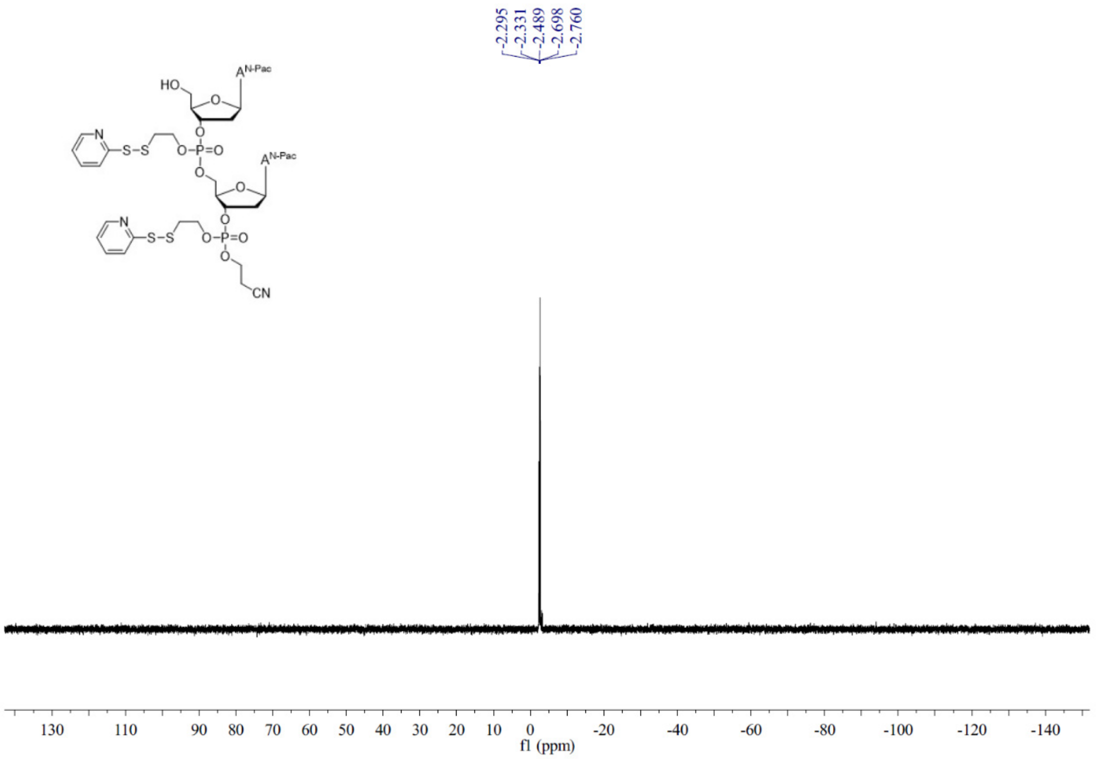
-2.295
-2.331
-2.489
-2.698
-2.760
HO
A^N-Pac
CN
f1 (ppm)

^{1}H, ^{13}C, ^{31}P NMR spectra of **8**:

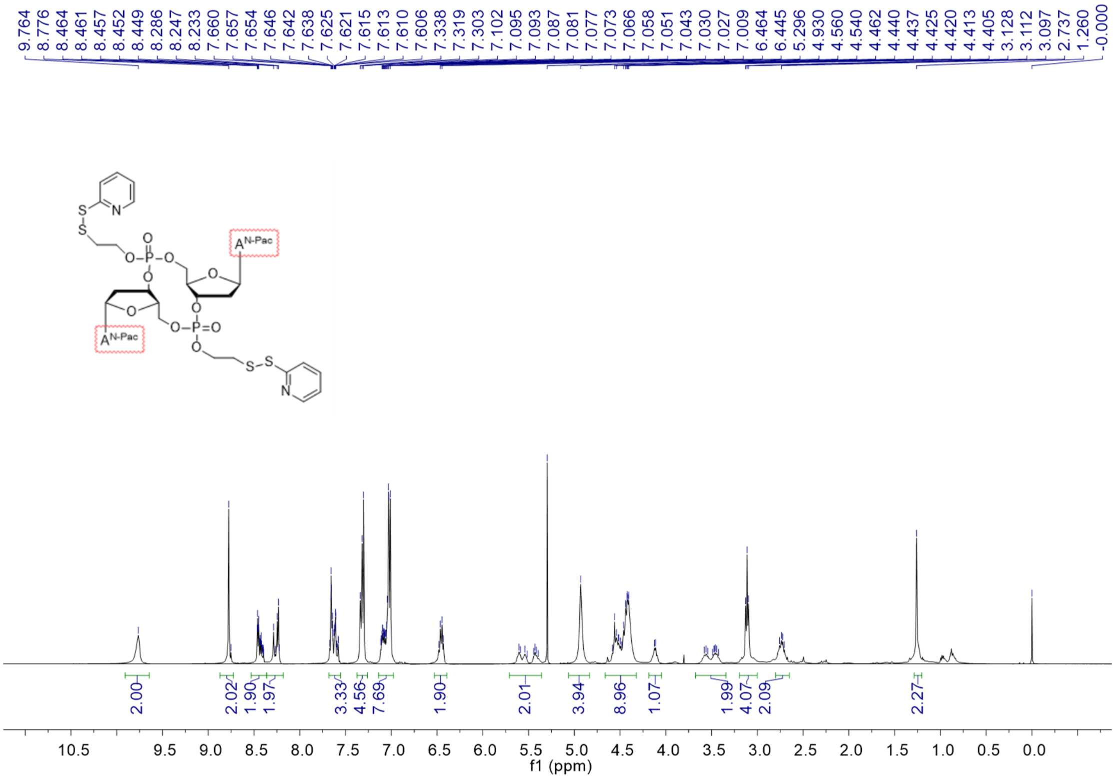

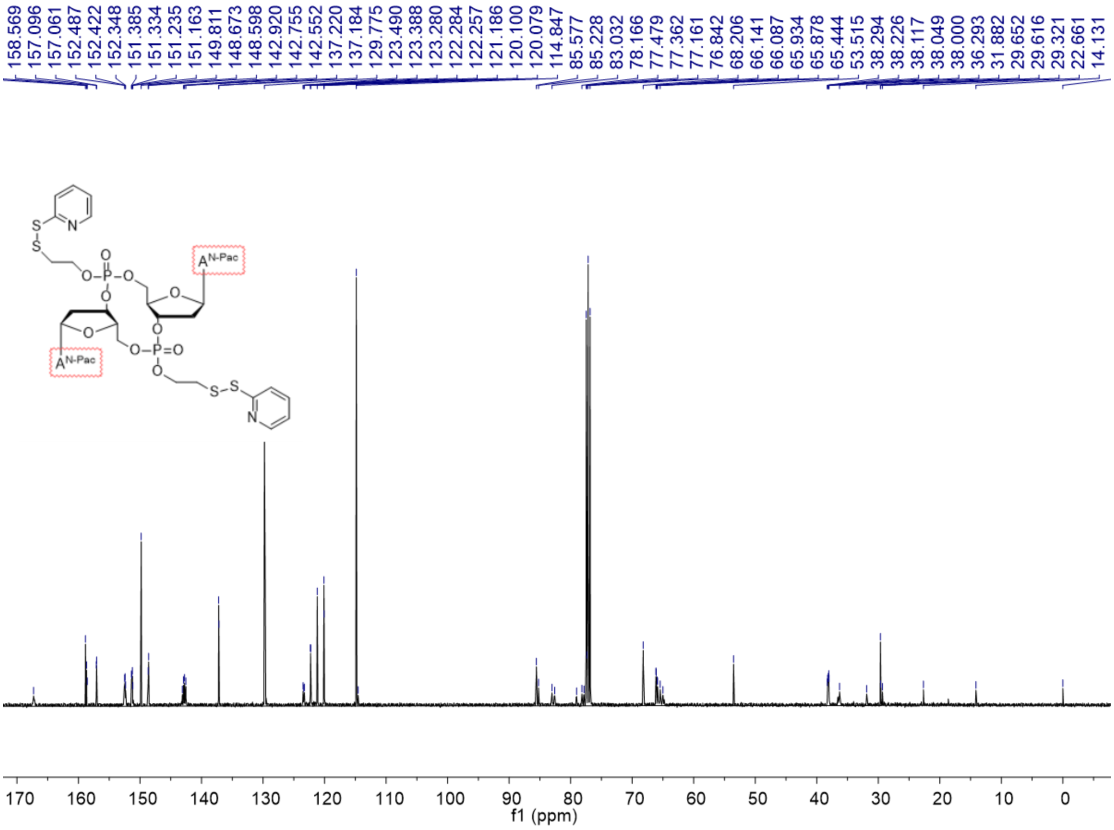

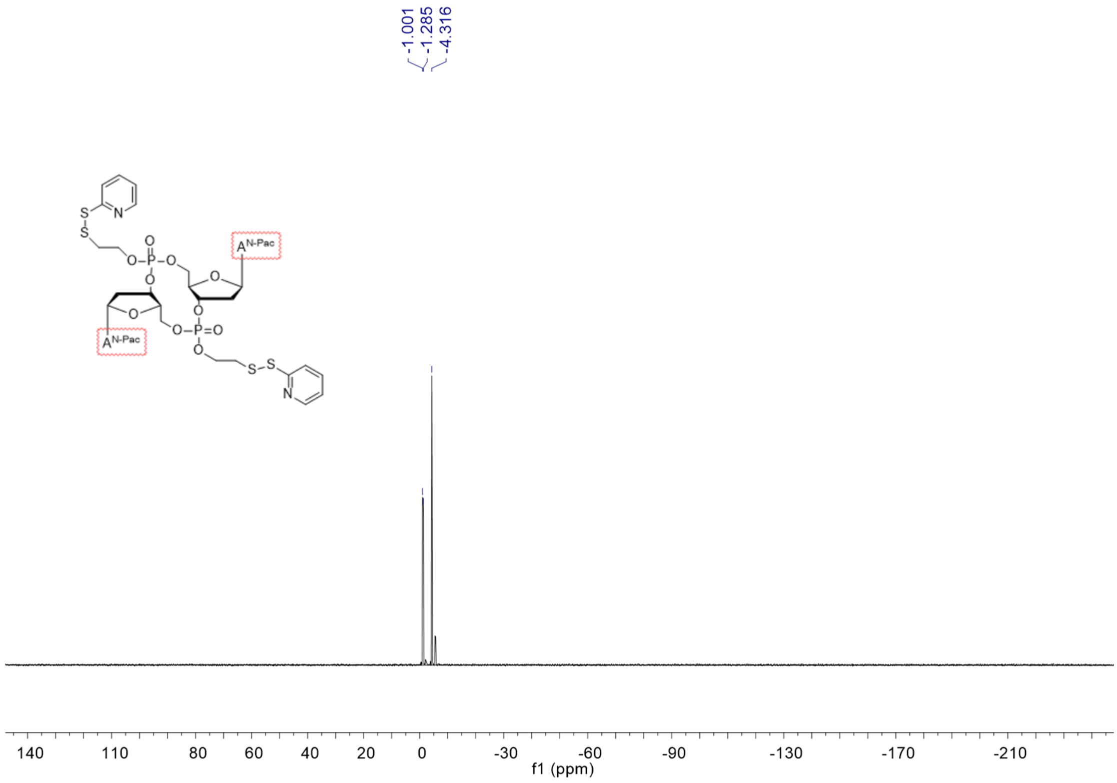

^{1}H, ^{13}C, ^{31}P NMR, HPLC spectra of **9**：

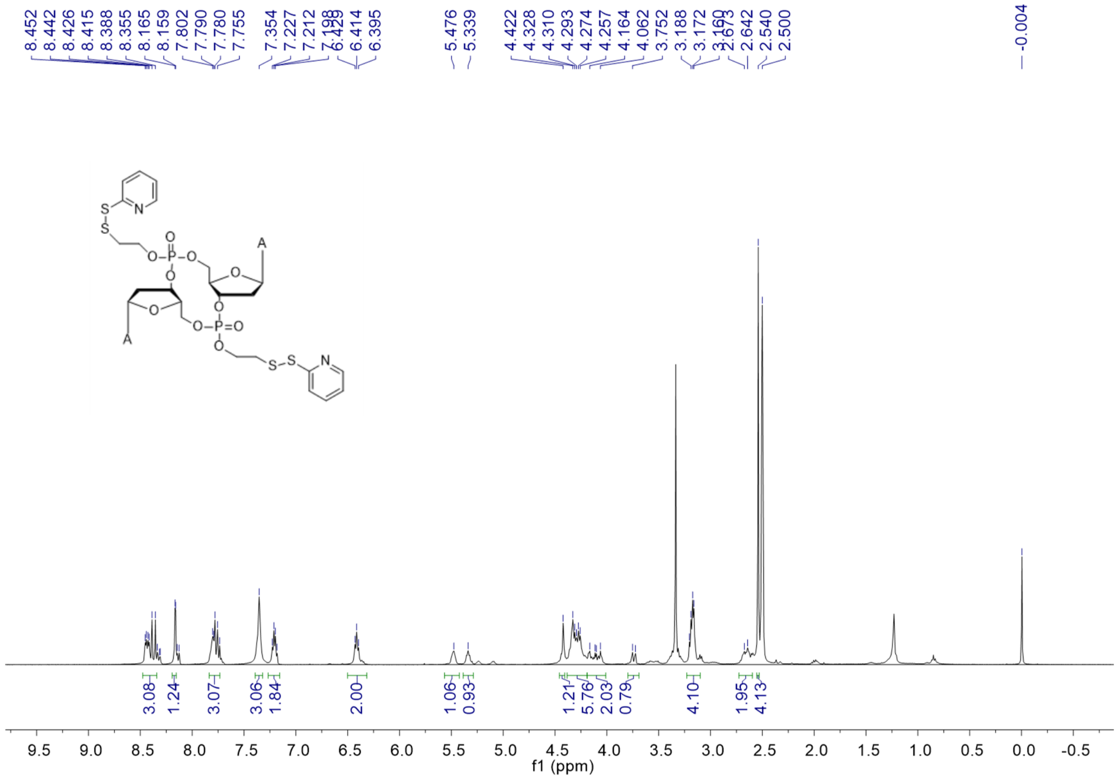

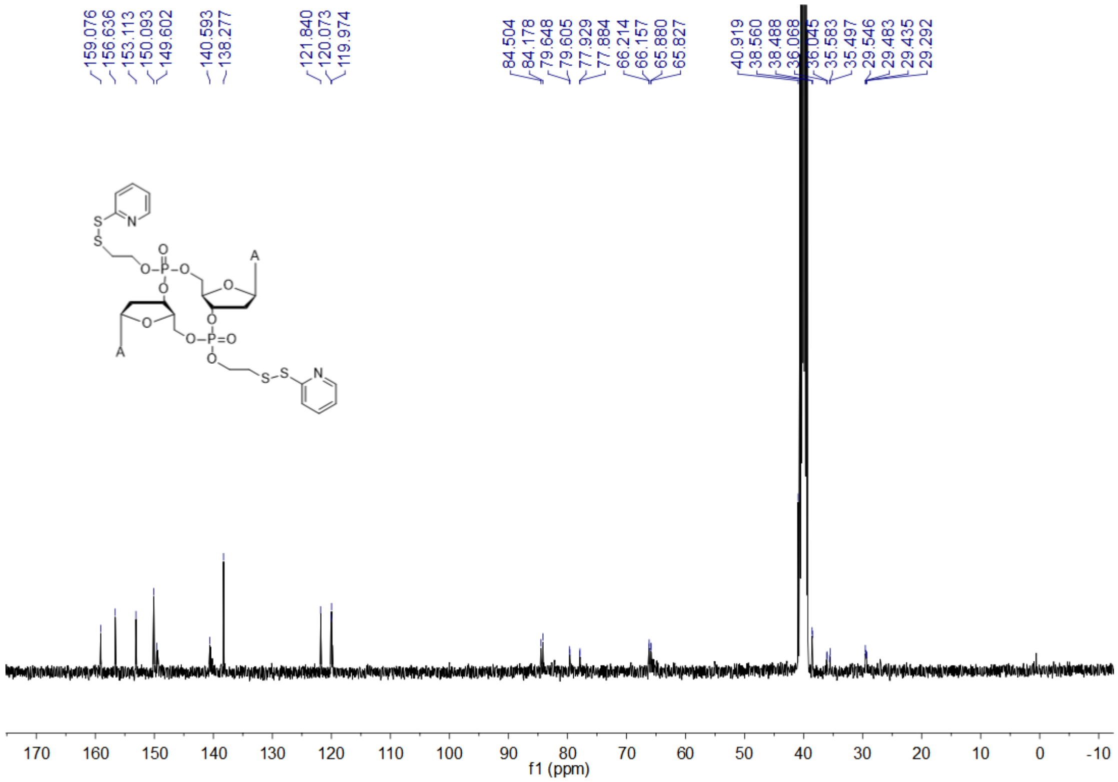
159.076
156.636
153.113
150.093
149.602
140.593
138.277
121.840
120.073
119.974
84.504
84.178
79.648
79.605
77.929
77.884
66.214
66.157
65.880
65.827
40.919
38.560
38.488
36.068
36.045
35.583
35.497
29.546
29.483
29.435
29.292
f1 (ppm)

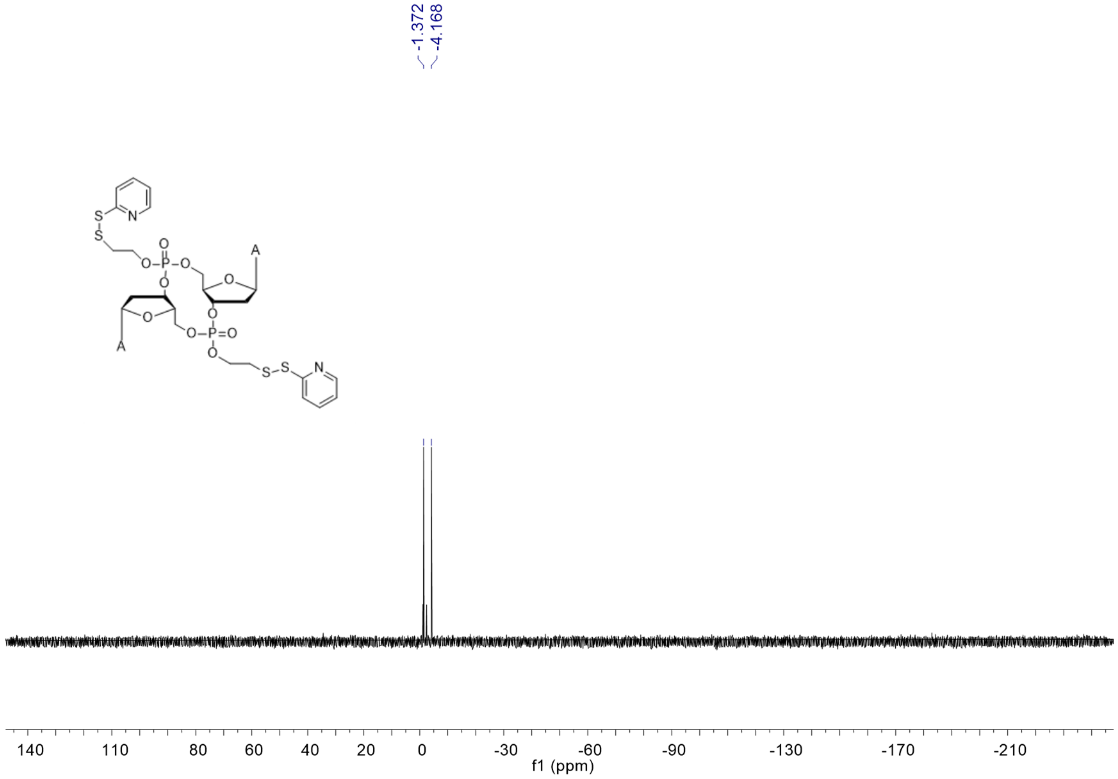
-1.372
-4.168
f1 (ppm)

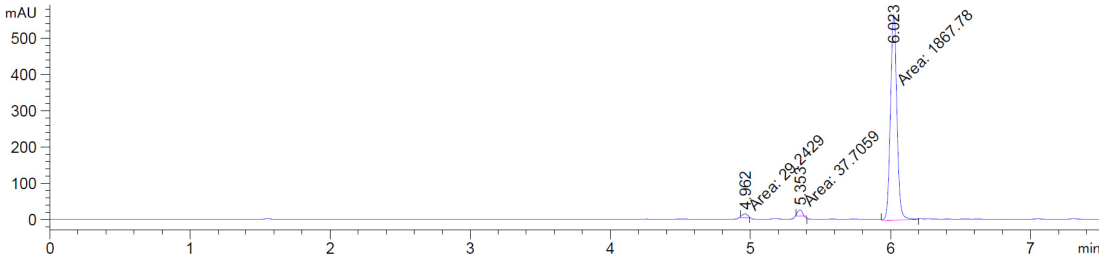

Wavelength = 254 nm

Peak #	RetTime [min]	Type	Width [min]	Area [mAU*s]	Height [mAU]	Area %
1	4.962	MM	0.0438	29.24292	11.12807	1.5115
2	5.353	MM	0.0382	37.70586	16.43100	1.9489
3	6.023	MM	0.0544	1867.78027	572.05725	96.5396

Raw Images of western blots:

pSTING

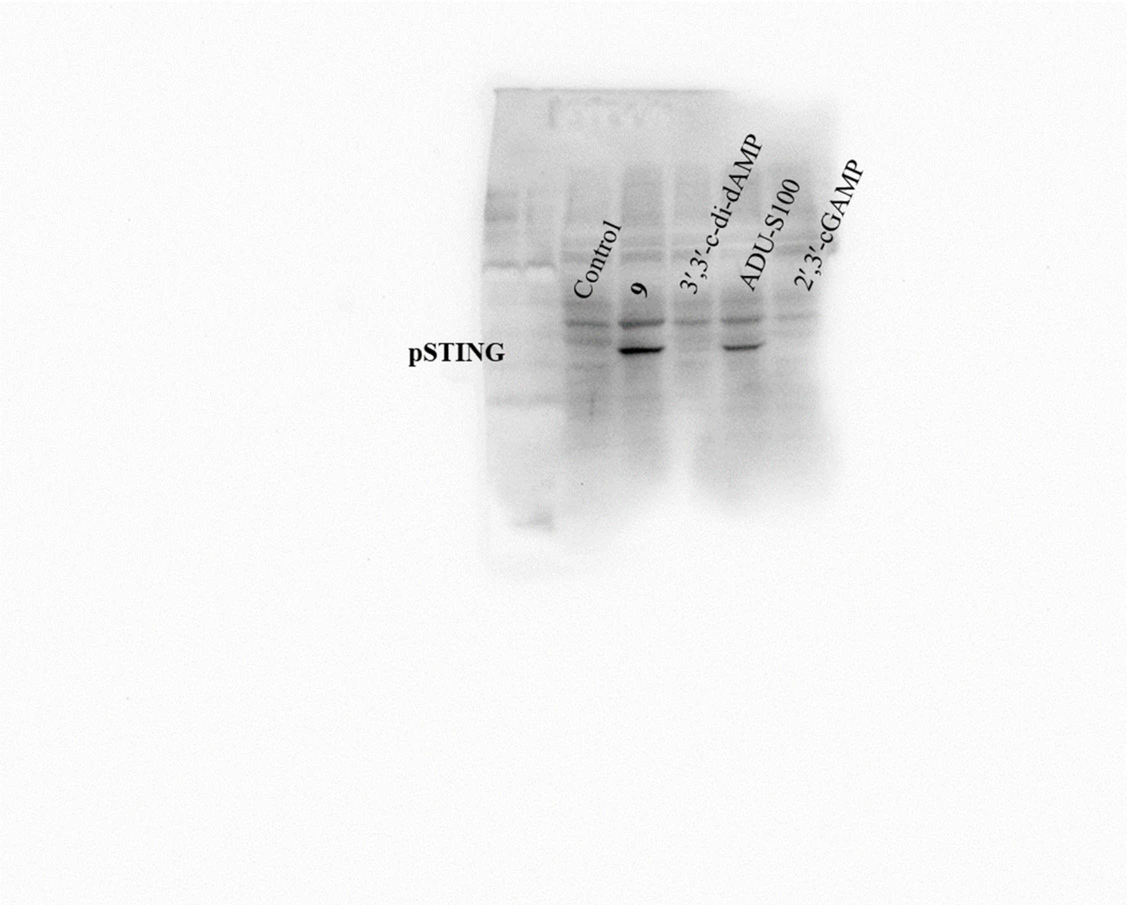

Total STING

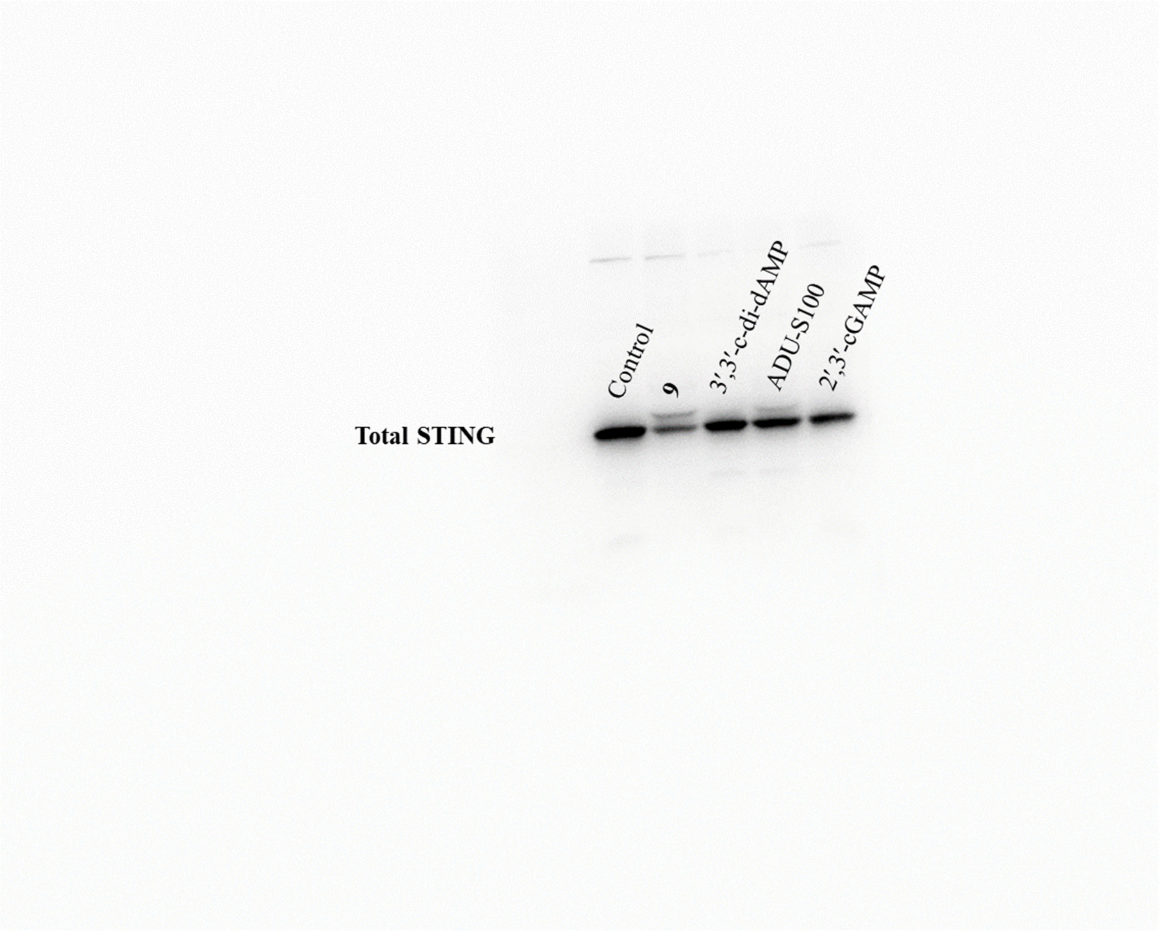

pTBK1

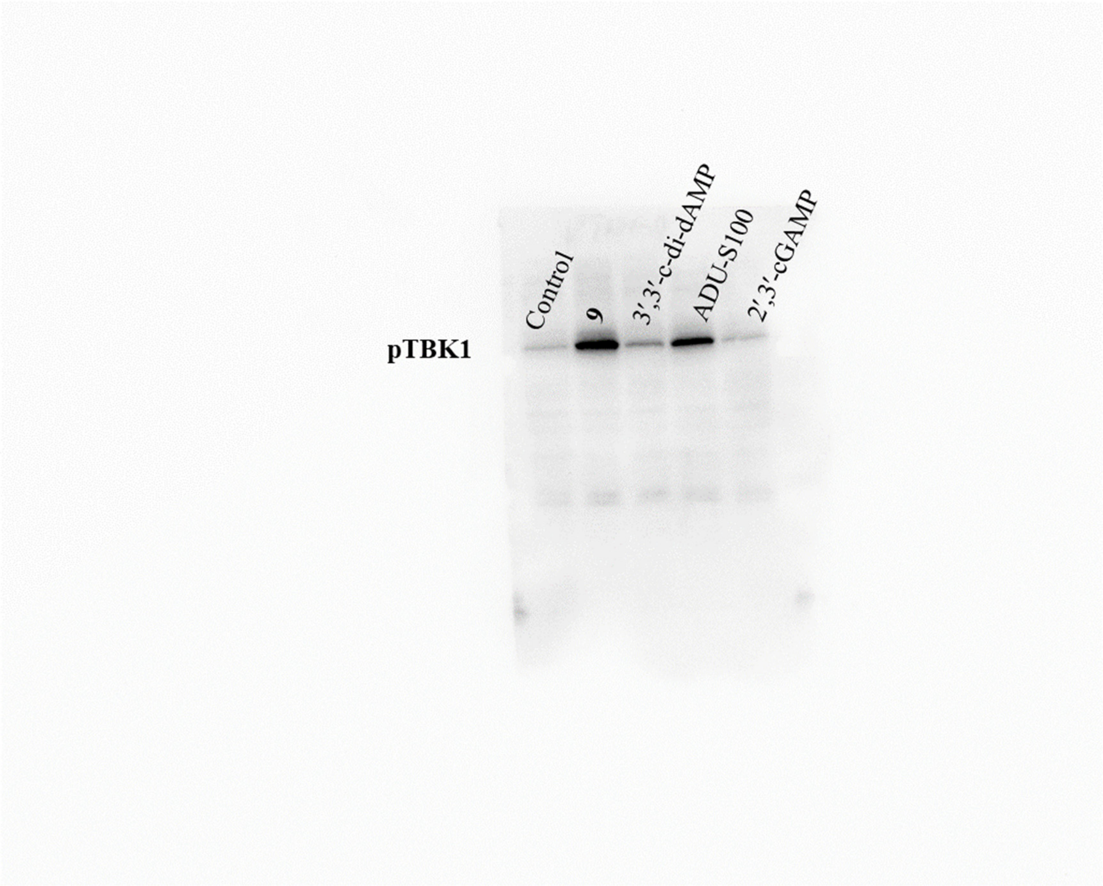

Total TBK1

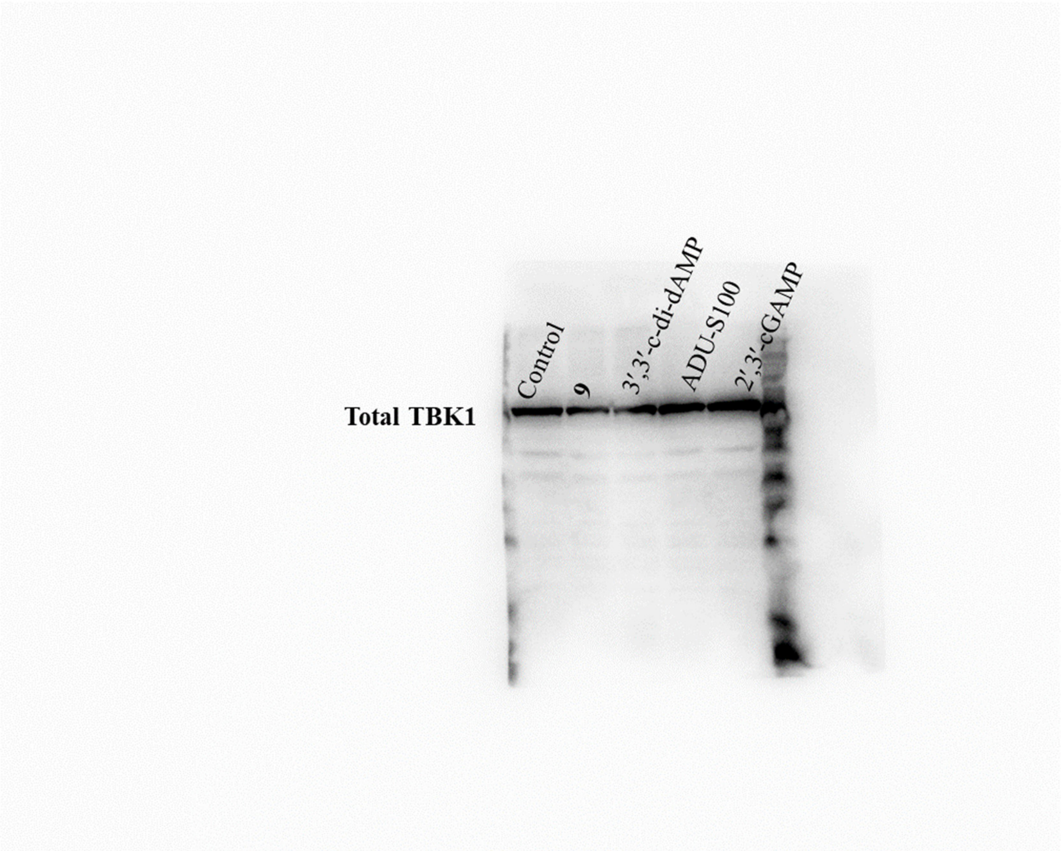

pIRF3

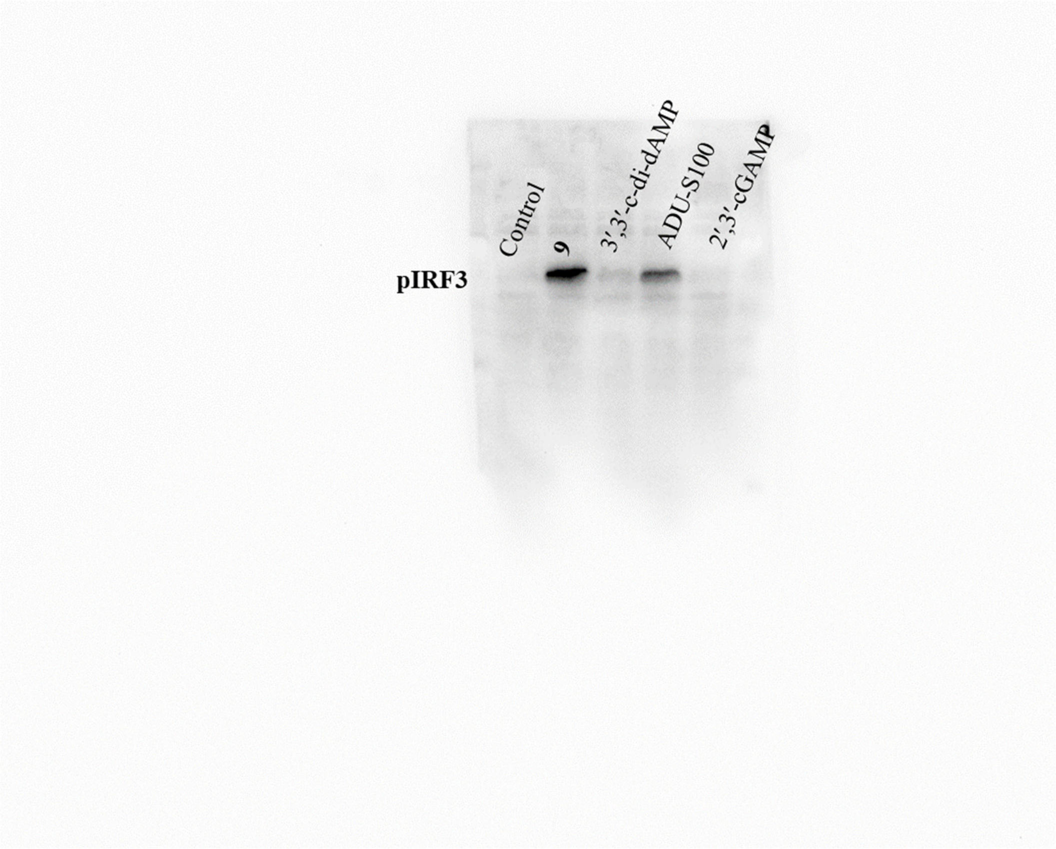

Total IRF3

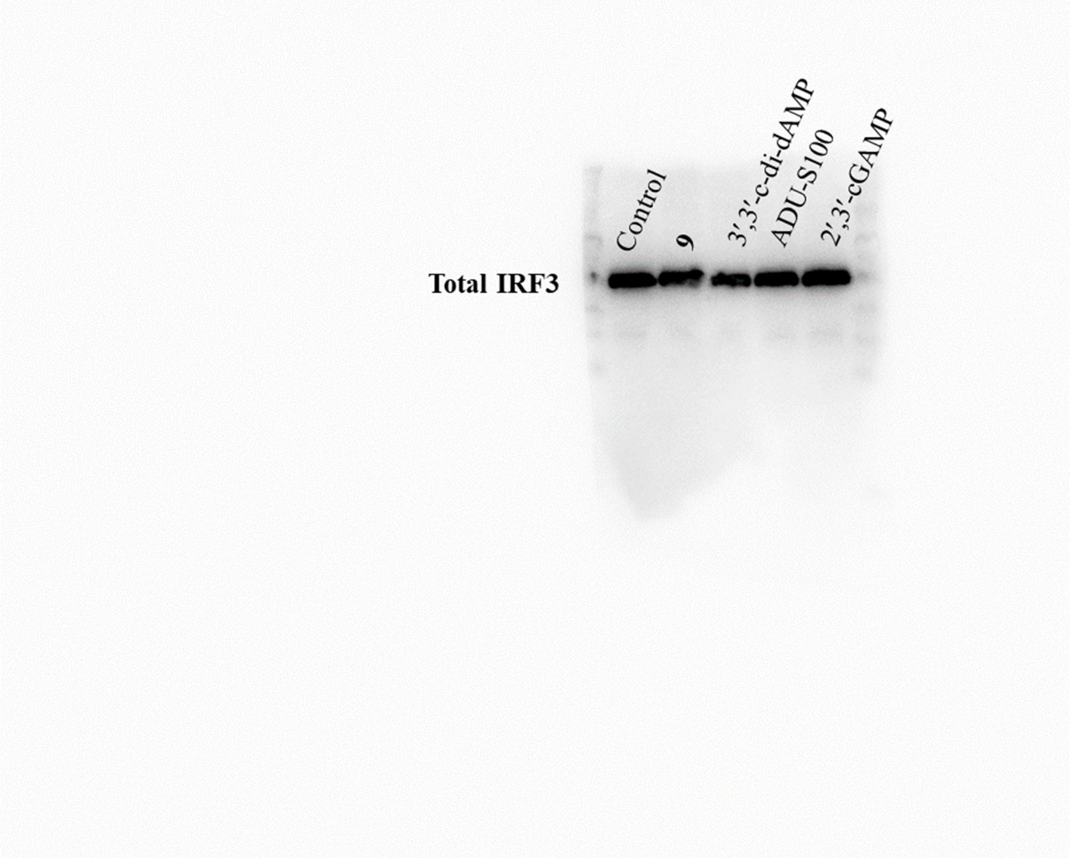

β-actin

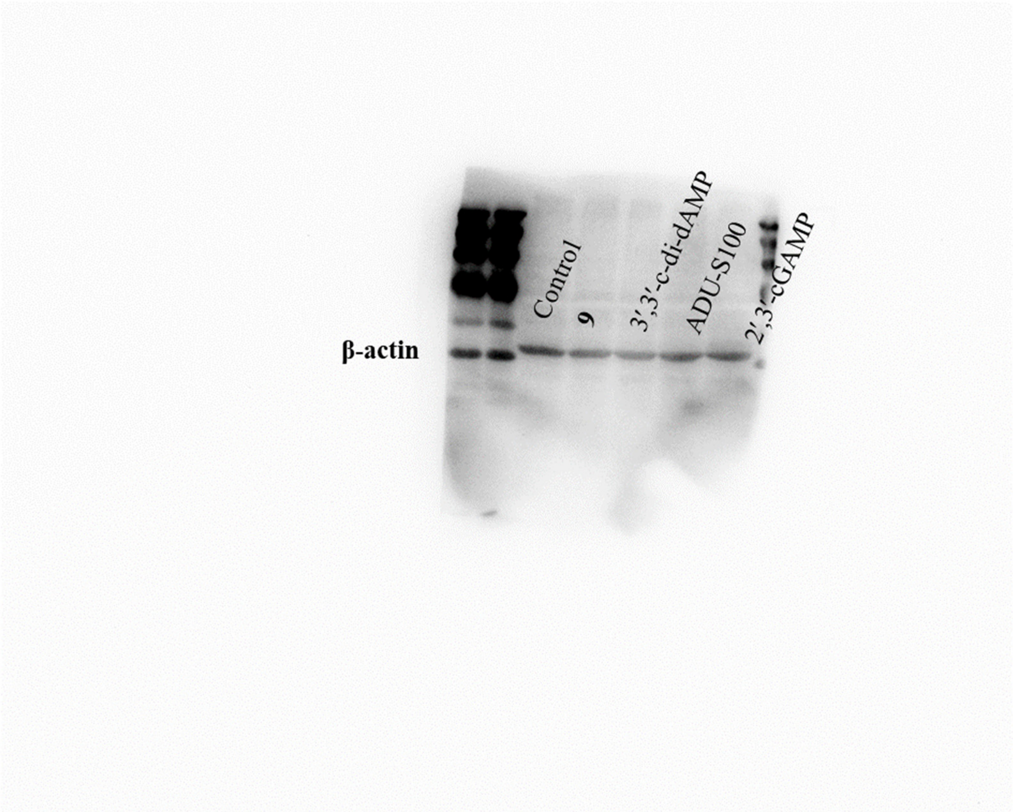

Supplement: Supplementary file 1 [file ijms-25-00086-s001.zip › ijms-2773104-supplementary.pdf]
